# A Systematic Review and Meta-Analysis of the Safety and Efficacy of 0.25% Lotilaner Ophthalmic Solution in the Treatment of Demodex Blepharitis

**DOI:** 10.7759/cureus.52664

**Published:** 2024-01-21

**Authors:** Bakhtawar Awan, Mohamed Elsaigh, Areej Tariq, Mohammed Badee, Abhinav Loomba, Yahya Khedr, Ahmed Abdelmaksoud

**Affiliations:** 1 Department of General and Emergency Surgery, Northwick Park Hospital, London, GBR; 2 Department of Opthalmology, Shaikh Zayed Hospital, Lahore, PAK; 3 Department of Ophthalmology, Perfect Vision Eye Hospital, Cairo, EGY; 4 Department of Opthalmology, Hull University Teaching Hospitals NHS Trust, Hull, GBR; 5 Department of Opthalmology, Hull University Teaching Hospitals NHS Trust, Cottingham, GBR

**Keywords:** collarettes, lotilaner ophthalmic solution, treatment of demodex, demodex folliculorum, demodex infestation, demodex mite

## Abstract

Demodex blepharitis is marked by an excessive presence of Demodex mites on the eyelids, particularly in the lash follicles. While these microscopic mites are a natural component of the skin microbiota, their overabundance can lead to ocular complications. Symptoms associated with Demodex blepharitis include eyelid itching, inflammation, and ocular irritation. Our objective is to investigate Lotilaner as a potential treatment for Demodex blepharitis, assessing both the safety and efficacy of the ophthalmic formula in managing this disease. We conducted research in Web of Science, PubMed, Cochrane Library, and Scopus up to November 2023. The quality of studies was evaluated using the Cochrane Risk of Bias tool, and it was employed to evaluate the quality of evidence. Our meta-analysis was executed using Review Manager 5.4. We evaluated the safety and efficacy of Lotilaner ophthalmic solution with a concentration of 0.25%. The following outcomes were assessed: clinically meaningful reduction in collarette, collarette cure, composite cure, drop comfort, erythema cure, mite density, and mite eradication. In the case of dichotomous data, we used the risk ratio (RR) with a 95% confidence interval (CI). In our analysis, all included studies, comprising a total of 891 participants, consistently reported clinically meaningful reductions in collarettes. The findings were statistically significant, with Lotilaner demonstrating a substantially higher reduction compared to the vehicle group (RR = 3.09, 95% CI [2.65-3.60]; *P*-value < 0.0001). Notably, results for Drop Comfort outcomes were nonsignificant, indicating no discernible differences compared to the group that used the vehicle (RR = 1.03, 95% CI [0.98-1.07]; *P*-value = 0.26). However, both mite density and mite eradication outcomes exhibited significant improvements with Lotilaner in comparison to the vehicle (RR = 2.58, 95% CI [2.25-2.95]; *P*-value < 0.0001) and (RR = 3.80, 95% CI [2.88-5.01]; *P*-value < 0.0001). The Lotilaner ophthalmic solution at 0.25% showed superior efficacy over the vehicle in reducing collarettes, achieving complete mite eradication within six weeks, and significantly decreasing erythema in Demodex blepharitis. It demonstrated safety with no reported side effects compared to the vehicle. Direct comparative studies with alternative treatments are recommended for a comprehensive assessment of efficacy and safety.

## Introduction and background

Demodex blepharitis is a commonly underestimated eye condition associated with eyelid redness, inflammation, and itching, accompanied by an additional burning sensation in the eyes. Its prevalence tends to rise with age, affecting 13% of 3- to 15-year-olds, 69% of 31- to 50-year-olds, 84% by the age of 60, and reaching 100% after 70 years of age [1]. Demodex blepharitis occurs when mites infest the follicles of eyelashes [2]. Two distinct types of mites are recognized for infesting the human body: Demodex folliculorum and Demodex brevis. They primarily inhabit areas around the eyelashes, hair follicles, and meibomian glands [3]. D. folliculorum mites feed on sebum and epithelial cells of the lid margin, resulting in epithelial hyperplasia and hyperkeratinization. This process led to the development of collarettes, which is also known as cylindrical dandruff, which is considered the characteristic indication of Demodex blepharitis [4]. These mites consist of decomposed (and occasionally living) mites, unprocessed elements, eggs of mites, and keratinized cells [5-7]. Demodex infestation causes harm through three different mechanisms: chemical, bacterial, and mechanical. This harm is a direct result of their presence in lash follicles [2]. Furthermore, it serves as a carrier for different types of bacteria, inducing inflammation through a hypersensitive reaction with the substances released by both the mites and the associated bacteria [2,8].

Demodex blepharitis is associated with various clinical indicators, including disruptions in tear film, dysfunction of meibomian glands, redness along the lid margin, loss of eyelashes, and, less commonly, corneal vascularization and corneal opacity [9,10]. In previous studies, the reported prevalence of ocular Demodex is documented to range from 16% to 70% in the general population [11]. As age increases, the infestation rate rises, reaching 84% in individuals aged 60 and peaking at 100% in those surpassing 70 years [9,12,13]. Ophthalmic professionals can easily identify concerns using a lamp focused on the lid margin, instructing patients to bend downward to facilitate an unobstructed assessment of the base of the lashes [14]. Patients frequently report symptoms such as eyelid itching, sensations of dryness, and ocular irritation. Approximately 80% of individuals affected by the disease note an adverse effect on their daily activities [15]. In the advanced stages of Demodex blepharitis, patients may experience recurrent chalazion, peripheral corneal vascularization, and corneal opacities [16]. Contemporary management involves practices such as lid scrubs, the application of warm compresses, the utilization of shampoos or products designed for lid hygiene, and mechanical extraction of the lid margin [2]. While these approaches may provide temporary alleviation of symptoms, there is no definitive evidence proving their ability to completely eradicate the presence of the mites and provide a permanent solution for the disease.

Previously, there were no treatments approved by the U.S. Food and Drug Administration (FDA) for Demodex blepharitis. Research has explored the use of systemic antiparasitics or antiprotozoal agents, as well as a combination of these treatment options. Additionally, various concentrations of local tea tree oil (TTO) ranging from 3% to 100% have been investigated [7,17-19]. However, there is a scarcity of studies utilizing controlled designs to determine the effectiveness of the agents addressing ocular Demodex, and a number of them are associated with notable adverse event profiles. Notably, terpinene 4-ol (T4O), the demodicidal component found in TTO, has recently been identified as detrimental to meibomian gland cells [20]. Therefore, the lack of a universally acknowledged standard for an efficacious treatment contributes to the ongoing difficulties faced by clinicians in identifying and their hesitancy to manage Demodex blepharitis.

The drug Lotilaner is a substance that causes spastic paralysis and demise in Demodex mites by inhibiting the parasites’ gamma-aminobutyric acid chloride channels without causing any harm to humans [21]. The initial approval was granted for veterinary use as an oral formulation in many different countries [22]. Recent trials indicate that the ophthalmic solution of Lotilaner at 0.25% possesses topical treatment qualities, showing promise in eradicating Demodex mites and prolonging the elimination of collarettes with a cure for eyelid redness [21]. In our meta-analysis, we are investigating safety and efficacy outcomes associated with the use of Lotilaner solution in the eyes for the management of Demodex blepharitis.

## Review

Methodology

We conducted our systematic review and meta-analysis in accordance with the latest revisions of the Preferred Reporting Items for Systematic Reviews and Meta-Analyses (PRISMA) statement and the Cochrane guidelines [23,24].

Literature Search and Data Collection

Our research was conducted until November 3, utilizing four databases: PubMed, Cochrane Library, Scopus, and Web of Science. Our research terms and strategy were ("Demodex Blepharitis" OR "Demodex-associated blepharitis") AND (Lotilaner OR Credelio OR Xdemvy OR "TP-03").

Studies Selection and Eligibility Criteria

We included randomized controlled trials (RCTs) based on the following criteria: 1) patients with Demodex blepharitis; 2) intervention: Lotilaner; 3) comparator: control, placebo, or vehicle; 4) outcomes: clinically meaningful collarette reduction, collarette cure, composite cure, drop comfort, erythema cure, mite density, and mite eradication. Following the completion of the research, the initial step involved the elimination of duplicates using EndNote. Subsequently, we conducted screening based on titles and abstracts, followed by a comprehensive review of full texts according to our eligibility criteria. Additionally, the references underwent examination by two reviewers to identify any potentially overlooked relevant articles. These screening steps were carried out by two authors.

Quality Assessment

Our included studies underwent assessment with version 1 of the Cochrane Risk of Bias tool [25]. This tool comprises the following domains: (1) selection bias; (2) allocation of arms; (3) blinding of participants and investigators; (4) assessment of blinding of the outcomes; and (5) randomization of the included population. Bias judgment in each domain can be categorized as high, low, or unclear risk of bias.

Data Extraction

Our data were retrieved in an Excel sheet, containing the following: baseline and summary characteristics, including study ID, study arms, site of the studies, age, gender, follow-up duration, inclusion criteria characteristics of the selected population, primary outcome, and conclusion.

Data Synthesis

Data synthesis was conducted using Review Manager (RevMan) software version 5.4. For dichotomous data, we presented risk ratios (RRs) and their corresponding 95% confidence intervals (95% CIs). Heterogeneity was assessed using the *I*-square test (*I*^2^) and the chi-square test, with significance determined by a *P*-value < 0.5. Studies were deemed heterogeneous if the chi-square *P*-value was <0.1 and the *I*^2^ value exceeded 50%. In cases of heterogeneity, we utilized the random-effects model for pooling data, whereas the fixed-effects model was employed for homogeneous data.

Meanings of the Outcomes

Mite eradication: This outcome, measured as 0 mites per lash, denotes the elimination or substantial reduction in the population of Demodex mites on the eyelids, specifically in the lash follicles. The assessment of mite eradication serves as an indicator of the improvement in clinical symptoms.

Mite density: This refers to the total number of mites present in one eye or on the eyelid. The assessment of mite density reduction is often an important aspect of evaluating the effectiveness of treatments for Demodex blepharitis.

Composite cure: In the context of Demodex blepharitis, it typically refers to an overall improvement or resolution of multiple symptoms associated with the condition, such as eyelid itching, inflammation, and ocular irritation.

Clinically meaningful collarette reduction: This term denotes the reduction of the number of collarettes to ten or fewer (grade 0 or 1) [26].

Drop comfort: This term describes the comfort experienced when eye drops are instilled into the eye.

Erythema cure: Achieving grade 0 erythema is considered a cure for erythema, measured by the change in erythema score from baseline [26].

Results

Literature Search and Study Selection

**Figure 1:**
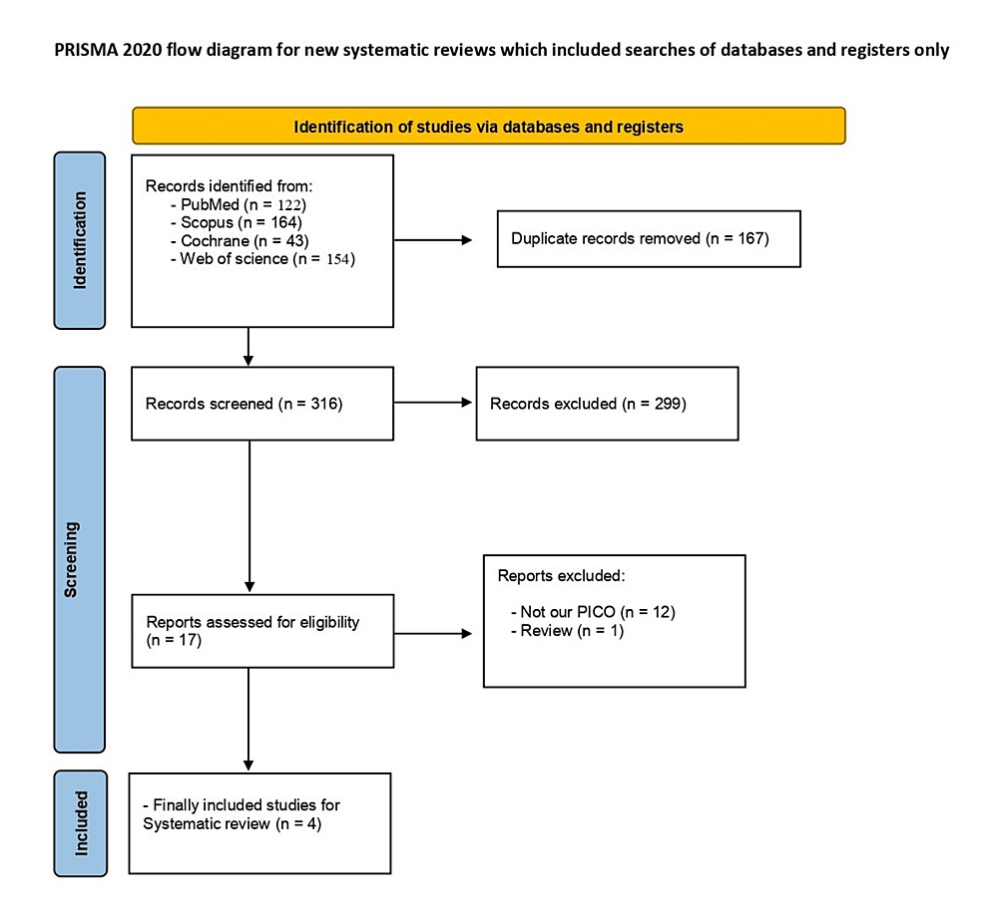
PRISMA flow diagram. PRISMA, Preferred Reporting Items for Systematic Reviews and Meta-Analyses; PICO, Patient/Population, Intervention, Comparison, and Outcome

Following our search strategy, we identified 316 articles after the removal of duplicates. Subsequent title and abstract screening narrowed down the selection to 17 studies for full-text screening. Finally, four studies [27-30] fulfilled our inclusion criteria and were deemed suitable for quantitative and qualitative analysis. The complete detailed PRISMA flowchart is shown in Figure 1.

Study Characteristics

The four studies included in our analysis were exclusively RCTs, with a collective participant pool totaling 947 individuals. There is a higher percentage of females than males in the study population. The majority of participants were aged over 60 years. The follow-up duration across three studies was 1.5 months, while the fourth study had a follow-up period less than the other studies. The inclusion criteria across the studies exhibited uniformity. All the included studies had the same location as they were conducted in the United States. The baseline and summary characteristics of all the included RCTs are presented in Table 1.

**Table 1:** Baseline and summary. Sources: [27-30].

Study ID		Country	Study design	Study arms, *n*	Male, *N* (%)	Age, Mean ± SD (years)	Follow-up duration	Inclusion criteria	Primary outcomes	Conclusions
Gaddie et al. (2023) [27]	Lotilaner ophthalmic solution at conc. 0.25%	United States	Randomized, double-masked, vehicle-controlled, multicenter, phase 3 clinical trial	203	106 (52.2)	63.9 ± 15.15	43 days	The eye with the higher mite density at the screening visit was considered for analysis. If both eyes had equal mite density, the right eye was the analysis eye.	Collarette cure, composite cure, erythema cure, and mite eradication	Twice-daily treatment with Lotilaner ophthalmic solution 0.25% for six weeks generally was safe and well tolerated and met the primary endpoint and all secondary endpoints for the treatment of Demodex blepharitis compared with vehicle control.
	Control			209	106 (50.7)	65.1±13.35				
Yeu et al. (2023) [30]	Lotilaner ophthalmic solution at conc. 0.25%	United States	Randomized, controlled, double-masked, phase 2b/3 clinical trial	212	89 (42.0)	66.1 6 ± 12.1	43 days	The eye with the higher mite density at the screening visit was considered the analysis eye. If both eyes had equal mite density, the right eye was the analysis eye.	Collarette cure, composite cure, and mite eradication	Twice-daily treatment with a novel Lotilaner ophthalmic solution, 0.25% for 43 days, is safe and effective for treating Demodex blepharitis compared with the vehicle control.
	Control			209	92 (44.0)	67.8 6 ± 12.6				
Yeu et al. (2022) [29]	Lotilaner ophthalmic solution at conc. 0.25%	United States	Randomized, controlled, double-masked clinical trial	27	NA	NA	42 days	Considering the analysis eye, if both eyes had equal mite density, the right eye was the analysis eye.	Collarette cure, composite cure, and mite eradication	Twice-daily, 42-day treatment with novel Lotilaner ophthalmic solution, 0.25%, is safe and effective for treating Demodex blepharitis compared to the vehicle control.
	Control			27	NA	NA				
Gonzalez-Salinas et al. (2022) [28]	Lotilaner ophthalmic solution at conc. 0.25%	United States	Phase II, randomized, controlled, double-masked clinical trial	30	NA	NA	28 days	The eye with the higher mite density during analysis is designated as the analysis eye.	Collarette cure, composite cure, and mite eradication	For Demodex blepharitis, treatment with Lotilaner ophthalmic solution, 0.25%, for four weeks is safe and effective. The improvement in collarette grade and mite density observed during the treatment period persisted for at least two months following treatment cessation.
	Control			30	NA	NA				

The Quality of the Included Studies

**Figure 2:**
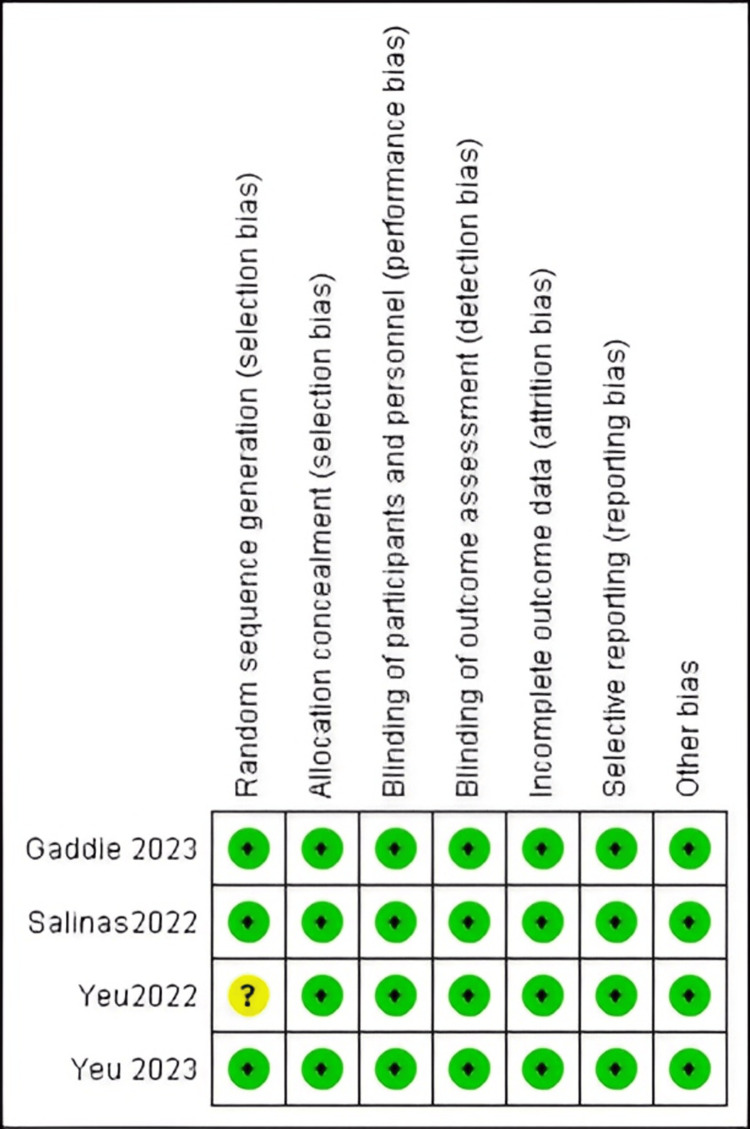
Risk-of-bias summary Sources: [27-30].

All the included studies were of high quality and exhibited low bias across all outcomes, except for studies by Yeu et al. [29], which had an unclear risk of bias in one aspect. The details are presented in Figure 2.

Outcomes

**Figure 3:**
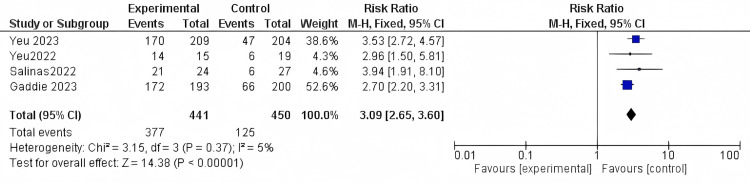
Clinically meaningful collarette reduction. Data have been represented as numbers and percentages. Sources: [27-30]. CI, confidence interval

Clinically meaningful collarette reduction: All the included studies reported the clinically meaningful collarette reduction outcome with 891 participants. The results were significant, as Lotilaner showed a higher reduction when compared to the vehicle (RR = 3.09, 95% CI [2.65 to 3.60]; *P*-value < 0.0001). Results were homogenous (*I*^2^ = 5%; *P *= 0.37) (Figure 3).

**Figure 4:**
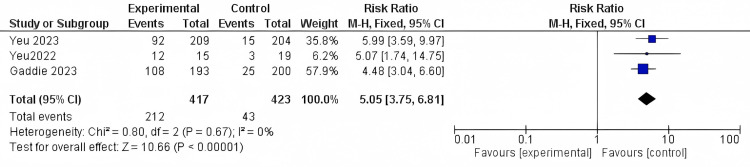
Collarette cure. Data have been represented as numbers and percentages. Sources: [27,29,30]. CI, confidence interval

Collarette cure: Three different studies reported the Collarette cure outcome, involving a total of 840 patients. The results were significant as(RR = 5.05, 95% CI [3.75 to 6.81]; *P*-value < 0.0001). The results were found to be homogenous (*I*^2^ = 0%; *P* = 0.67) (Figure 4).

**Figure 5:**
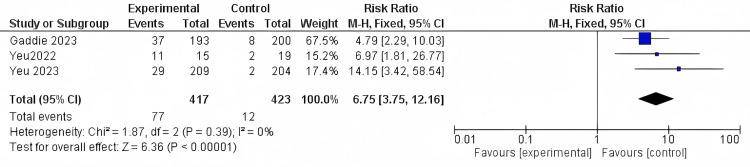
Composite cure. Data have been represented as numbers and percentages. Sources: [27,29,30]. CI, confidence interval

Composite cure: Three different studies reported the composite cure outcome with 840 participants. The results were significant (RR = 6.75, 95% CI [3.75 to 12.16]; *P*-value < 0.0001). The results were homogenous (*I*^2^ = 0%; *P* = 0.67) (Figure 5).

**Figure 6:**
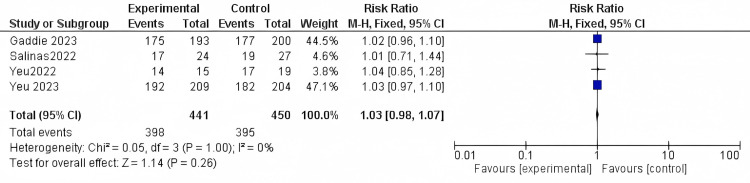
Drop comfort. Data have been represented as numbers and percentages. Sources: [27-30]. CI, confidence interval

Drop comfort: The included studies reported the drop comfort outcome with 891 participants. The results were nonsignificant, as there were no differences compared to the control (RR = 1.03, 95% CI [0.98 to 1.07]; *P*-value = 0.26). The results were homogenous in the fixed-effect model (*I*^2^ = 0%; *P* = 1) (Figure 6).

**Figure 7:**
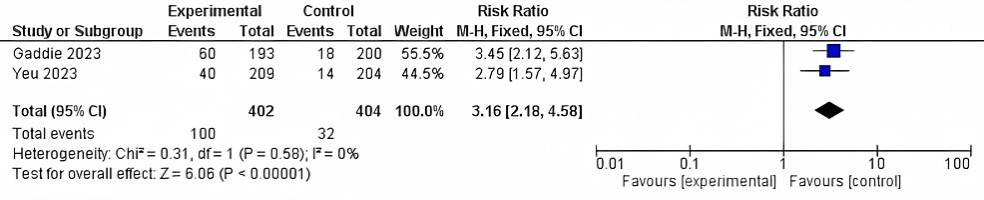
Erythema cure. Data have been represented as numbers and percentages. Sources: [27,30]. CI, confidence interval

Erythema cure: Only two studies reported erythema cure outcomes with 806 patients. The results were significant as the Lotilaner solution showed a higher rate of cure (RR = 3.16, 95% CI [2.18 to 4.58]; *P*-value < 0.0001). The results were homogenous (*I*^2^ = 0%; *P *= 0.58) (Figure 7).

**Figure 8:**
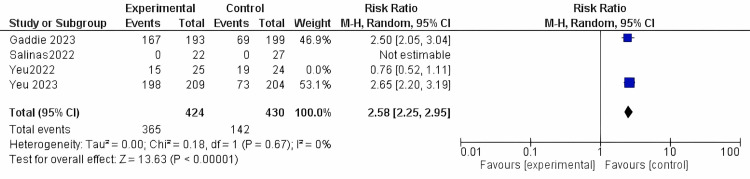
Mite density. Data have been represented as numbers and percentages. Sources: [27-30]. CI, confidence interval

Mite density: This outcome was reported in the four studies included, encompassing a total population of 854 participants. The intervention could significantly reduce the mite density in comparison to the vehicle (RR = 2.58, 95% CI [2.25 to 2.95]; *P*-value < 0.0001). The results were homogenous in the random model (*I*^2^ = 0%; *P *= 0.67) (Figure 8).

**Figure 9:**
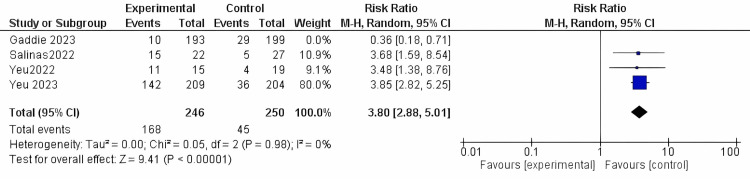
Mite eradication. Data have been represented as numbers and percentages. Sources: [27-30]. CI, confidence interval

Mite eradication: This outcome was reported in all of the included studies, encompassing a total population of 496 participants. The intervention could significantly achieve mite eradication in comparison to the vehicle (RR = 3.80, 95% CI [2.88 to 5.01]; *P*-value < 0.0001). The results were homogenous in the random model (*I*^2^ = 0%; *P* = 0.98) (Figure 9).

Discussion

We assessed the safety and efficacy of Lotilaner in the form of an ophthalmic solution with a concentration of 0.25%. In our study, the intervention was superior to the vehicle (control) in all the outcomes and many significant differences were noticed. The clinically meaningful collarette reduction was significantly greater in Lotilaner, approximately three times that of the control. The collarette cure incidence was also higher than the control. The Lotilaner ophthalmic solution of 0.25% could achieve both the composite cure and the erythema cure while no significance was noted in the drop comfort. Both mites eradication and mite density decrease were significantly achieved in the treatment of Demodex blepharitis. The extent of collarettes serves as an indicator of the intensity of Demodex mite infestation, with higher severity of collarettes correlating to a more pronounced and severe presence of these mites [31,32]. However, it's noteworthy that all the patients were at the same grade of infection before the start of the treatment. The elimination of mites closely paralleled the disappearance of collarettes, confirming within this study the causal relationship between the substantial number of Demodex mites and this significant indicator. The Lotilaner ophthalmic solution at 0.25% was generally well-tolerated in adults with Demodex blepharitis, exhibiting a safety profile comparable to its vehicle when the study drug was applied for a duration of up to a month and a half.

Lotilaner marks the first approved groundbreaking antiparasitic treatment for Demodex blepharitis, a prevalent condition affecting the lid margin and significantly impacting patients on a psychological level. It can completely cure the disease with a high safety profile. In a different investigation, a mite eradication rate of 36% was documented when a low-concentration (7.5%) TTO shampoo was applied for four weeks to mitigate the potential adverse effects of TTO [7]. Consequently, the effectiveness of TTO in eliminating mites remains unclear [3]. There are various side effects for TTO in comparison to Lotilaner. The main side effects linked to TTO treatment include contact dermatitis, ocular irritation, and allergic reactions [7,33]. From the preceding sentences, we infer that the Lotilaner ophthalmic solution is deemed superior to TTO. However, it's worth noting that no previous studies have directly compared the two treatment options for safety or efficacy. Additionally, there is a lack of research regarding treatment strategies for Demodex mites.

Our study demonstrates notable strengths as the first systematic review and meta-analysis specifically designed to evaluate the safety and efficacy of treating Demodex blepharitis with the ophthalmic solution of Lotilaner at a concentration of 0.25%. All incorporated studies maintained a high level of quality, employing RCTs as the study design. This enhances the robustness of the findings and minimizes the risk of bias. However, certain limitations merit consideration. The number of the included studies and the total population is low due to the lack of research on the study. The relatively brief follow-up period employed may not be sufficient to elucidate the potential for Demodex mites to re-infest the eyelids post-eradication, prompting the need for extended follow-up investigations to ascertain the likelihood of migration from facial or other bodily regions. Furthermore, the included studies employed diverse methodologies for outcome assessment, introducing variability in the interpretation of results. Finally, our comparison of the Lotilaner solution was solely against control, and it is imperative for future research to conduct direct head-to-head comparisons with alternative treatment modalities to identify the most efficacious approach for Demodex mite eradication.

## Conclusions

We are currently conducting a pioneering meta-analysis to systematically compare Lotilaner ophthalmic solution and control in terms of efficacy for Demodex blepharitis. The Lotilaner ophthalmic solution at a concentration of 0.25% demonstrated superiority over the vehicle across several parameters associated with Demodex blepharitis. It exhibited enhanced efficacy in reducing collarettes, achieving complete mite eradication within a month and a half timeframe, and significantly decreasing instances of erythema. Furthermore, Lotilaner proved to be a safe option with no reported side effects in comparison to the vehicle. We recommend conducting direct comparative studies with alternative treatment modalities for a comprehensive evaluation of efficacy and safety.
